# The influence of equine body weight gain on inflammatory cytokine expressions of adipose tissue in response to endotoxin challenge

**DOI:** 10.1186/s13028-020-00515-5

**Published:** 2020-04-22

**Authors:** Dominique Blaue, Carola Schedlbauer, Janine Starzonek, Claudia Gittel, Walter Brehm, Matthias Blüher, Martin Pfeffer, Ingrid Vervuert

**Affiliations:** 1 Institute of Animal Nutrition, Nutrition Diseases and Dietetics, Leipzig University, An den Tierkliniken 9, 04103 Leipzig, Germany; 2 Department for Horses, Leipzig University, Leipzig, Germany; 3 Department of Medicine, Leipzig University, Leipzig, Germany; 4 Institute of Animal Hygiene and Veterinary Public Health, Leipzig University, Leipzig, Germany

**Keywords:** Adipose tissue, Equine, Inflammation, Lipid metabolism, Obesity

## Abstract

**Background:**

Human obesity is linked with systemic inflammation. However, it is still controversial if equines produce more inflammatory cytokines with increasing body weight and if the production of those show breed type specific patterns. The main objective of this study was to determine if diet induced obesity is associated with increased inflammatory signatures in adipose tissue of equines and if a breed predisposition exists between ponies and horses. Additionally, we aimed to identify adipose tissue depot differences in inflammatory cytokine expression. Nineteen healthy, non-overweight and metabolically healthy equines received a hypercaloric diet for 2 years. Body weight, body condition score and cresty neck score were assessed weekly throughout the study. At three time points, insulin sensitivity was determined by a combined glucose-insulin test. Adipose tissue samples were collected from two intra-abdominal and two subcutaneous depots under general anesthesia at each time point after an endotoxin trigger. In the adipose tissue samples levels of *CD68* mRNA (a marker of macrophage infiltration) and pro-inflammatory cytokine mRNA (*IL*-*1β*, *IL*-*6* and *TNFα*) were analyzed with RT-qPCR. As markers of lipid metabolism mRNA levels of lipoprotein lipase (LPL) and fatty acid binding protein 4 (FABP4) were determined with RT-qPCR.

**Results:**

*CD68* mRNA levels increased with body weight gain in several adipose tissue (AT) depots (Wilcoxon signed rank test with Bonferroni correction; retroperitoneal AT horses: P = 0.023, mesocolonial AT horses: P = 0.023, subcutaneous tail head AT ponies: P = 0.015). In both abdominal depots *CD68* mRNA levels were higher than in subcutaneous adipose tissue depots (Kruskal–Wallis-ANOVA with Bonferroni correction: P < 0.05). No breed related differences were found. Pro-inflammatory cytokine mRNA *IL*-*1β*, *IL*-*6* and *TNFα* levels were higher in subcutaneous depots compared to abdominal depots after body weight gain. *IL*-*1β, IL*-*6* and *TNFα* mRNA levels of mesocolon adipose tissue were higher in obese horses compared to obese ponies (Mann–Whitney-U test; IL-1β: P = 0.006; IL-6: P = 0.003; TNFα: P = 0.049). In general, horses had higher *FABP4* and *LPL* mRNA levels compared to ponies in neck AT and tail AT at all time points.

**Conclusion:**

Our findings suggest an increased invasion of macrophages in intra-abdominal adipose tissue with increasing body weight gain in equines in combination with a low dose endotoxin stimulus. This might predispose equines to obesity related comorbidities. In obese horses mesocolon adipose tissue showed higher inflammatory cytokine expression compared to obese ponies. Additionally, subcutaneous adipose tissue expressed more pro-inflammatory cytokines compared to intra-abdominal adipose tissue. Horses had higher *FABP4* and *LPL* mRNA levels in selected AT depots which may indicate a higher fat storage capacity than in ponies. The differences in lipid storage might be associated with a higher susceptibility to obesity-related comorbidities in ponies in comparison to horses.

## Background

Today one of the main challenges in medicine is an increasing incidence of obesity and dealing with its metabolic consequences in both humans and equines [1, 2]. Human metabolic syndrome is characterized by obesity, hypertension, and increased blood concentrations of triglycerides and glucose [3]. The equine metabolic syndrome (EMS) is determined by obesity, insulin dysregulation (ID) and a predisposition to laminitis [4]. A systemic inflammatory state in obese humans has been reported for several years [5–7]. In the equine there is an ongoing debate about the contribution of low-grade systemic inflammation associated with obesity [8–11]. Only few equine studies have been performed, evaluating the effect of a standardized body weight (BW) gain on inflammatory cytokine levels. Most published studies report inflammatory markers in single spot samples of obese and/or insulin dysregulated horses without documenting the duration of obesity and other relevant factors such as long-term ration formulation [9, 12–14].

Considering pro-inflammatory cytokines in equine obesity, the current data are controversial. Some authors described differences in circulating pro-inflammatory cytokine levels between obese and/or sick equines compared to healthy equines [9, 14]. Other studies did not find differences in inflammatory cytokines between obese and healthy equines [12, 13]. In humans it has been proven that abdominal (abd) AT is the major site of pro-inflammatory cytokine production compared to other AT depots [5, 15]. It is still unclear which AT depot is more active in inflammatory cytokine production in equines [16]. One of the most detrimental obesity-related comorbidities in equines is laminitis. However, it has been suggested that a trigger factor such as endotoxins may contribute to obesity related comorbidities such as laminitis [17–19].

Glucose metabolism is closely linked with that of lipids [2, 20]. Lipid transport protein expressions, e.g. lipoprotein lipase (LPL) and fatty acid binding protein 4 (FABP4), are altered in human obesity. The enzyme LPL catalyzes the hydrolysis of triacylglycerides in blood vessels and mediates the absorption of fatty acids [21]. Obese humans with Type II diabetes expressed higher LPL levels in AT compared to healthy lean individuals [22]. Increased LPL activities were reported in equine studies in the context of feeding high fat diets [23, 24]. To the authors knowledge the effect of equine obesity on LPL expression has not yet been described. FABP4 is mainly expressed in AT and mediates the transport of fatty acids within adipocytes [25]. In human obesity studies FABP4 expression was down-regulated in AT, which may explain excessive fat uptake by other tissues such as the liver [26]. To the authors knowledge studies about FABP4 with increasing obesity in equines have not been published.

The present investigation was performed to identify the relationship of BW gain with inflammatory cytokine levels, macrophage invasion and lipid transport protein expressions in different AT depots of ponies and horses after a moderate endotoxin trigger. We hypothesized that there would be an increase of inflammatory markers in AT of equines within 2 years of excessive energy intake and BW gain with higher levels in ponies compared to horses. Furthermore, we hypothesized there would be higher inflammatory cytokine levels in sc AT compared to abd AT in obese equines. With respect to lipid metabolism we expected a down-regulation in LPL and FABP4 expression in AT with increasing obesity.

## Methods

### Animals

Ten Shetland pony geldings (mean age ± SD 6 ± 3 years) and nine Warmblood horse geldings (mean age ± SD 10 ± 3 years) owned by the Institute of Animal Nutrition, Nutrition Diseases & Dietetics, Leipzig University were included into this study. All equines had a moderate body condition score (BCS ≤ 3 out of 5) [27] without an increased cresty neck score (CNS ≤ 3 out of 5) [28] at the baseline (t0) of the study. The following exclusion criteria were defined in selection of the study equines: a previous history of ID, laminitis or pituitary pars intermedia dysfunction. All equines were housed in box stalls and were turned out daily onto a dry lot for approximately 5 h/day.

The project was approved by the Ethics Committee for Animal Rights Protection of the Leipzig District Government (No. TVV 32/15) in accordance with German legislation for animal rights and welfare.

### Study design

**Fig. 1:**
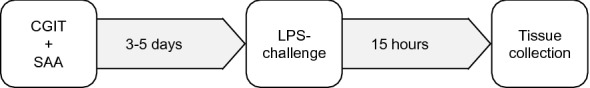
Protocol of sampling procedure at t0, t1 and t2

During the study the equines were fed a hypercaloric ration for 2 years. Basal AT and blood samples were obtained before starting the hypercaloric feeding protocol (t0; October, 15 to December, 11, 2015). Follow up AT and blood samples were taken after one (t1; October, 27 to December, 1, 2016) and two (t2; October, 13 to November, 30, 2017) years of feeding the hypercaloric diet. At each sampling point a combined glucose insulin test (CGIT) was performed. Three to five days after the CGIT, a lipopolysaccharide (LPS) challenge was conducted, and 15 h after LPS challenge tissue sampling was performed under general anaesthesia (Fig. 1). Samples of the abd AT samples were collected from the retroperitoneal AT (rp AT) at the margins of the incision and mesocolonial AT (mc AT) of the mesocolon descendens. Subcutaneous (sc) AT was obtained from the neck crest (neck AT) at the middle of the neck one to two cm ventral to the mane. Additionally, tail head adipose tissue (tail AT) was collected caudally from the croup lateral to the tail. During the BW gain period, weekly morphometric measurements (BW, BCS and CNS) were recorded.

### Feed management

*Acclimation period* Before t0 all equines had received a meadow hay ration that met maintenance energy requirement for metabolizable energy (ME) according to the Society of Nutrition Physiology (GfE) [29] for at least 2 weeks.

*Hypercaloric diet* The diet provided 200% of the maintenance energy requirements for ME [29]. Sixty percent of the energy was provided by meadow hay and 40% by a compound feed. Nutrient composition of the diet is shown in Additional file 1. Amounts of both feedstuffs were adjusted every 4 weeks to match the BW gain.

### Blood sampling

All blood samples were taken by venipuncture from the right or left jugular vein. Basal blood samples for serum amyloid A (SAA), insulin and glucose were taken after 8 h of fasting, between 7.00 and 8.00 a.m.. Subsequently, CGIT was performed according to Eiler et al. [30]. Serum tubes containing coagulation activator (Monovette, Sarstedt AG, Nuembrecht, Germany) were taken for insulin and SAA analysis. For glucose concentration, tubes containing sodium fluoride (S-Monovette, Sarstedt AG) were used. Serum tubes were centrifuged after 30 min of clotting time and sodium fluoride containing tubes were immediately centrifuged for 10 min at 865×*g.* Serum and plasma were removed, and aliquots were gradually frozen from − 20 to − 80 °C until analysis.

### LPS infusion

Three to five days after CGIT, a 14-gauge indwelling catheter (Milacath; Mila International, Florence, USA) was aseptically inserted in the jugular vein and 10 ng/kg BW LPS (*Escherichia coli* 055:B5; Sigma-Aldrich Chemie GmbH, Munich, Germany) diluted in 500 mL or1000 mL 0.9% saline, for the ponies and horses respectively, was infused over the course of 30 min. The animals were continuously monitored for at least 3 h after LPS challenge according to a modified pain score protocol [31] or until rectal temperature started to drop.

### AT sampling

Equines were sedated with 0.04 mg/kg BW romifidine (Sedivet, Boehringer Ingelheim Pharma GmbH & Co. KG, Ingelheim am Rhein, Germany) and 0.03 mg/kg BW butorphanol (Alvegesic, CP-Pharma Handelsgesellschaft GmbH, Burgdorf, Germany). General anaesthesia was induced with 0.08 mg/kg BW diazepam (Ziapam, Laboratoire TVM, Lempdes, France) and 3 mg/kg BW ketamine (Ursotamin, Serumwerk Bernburg AG, Bernburg, Germany). Animals were orotracheally intubated, and anaesthesia was maintained with isoflurane (CP-Pharma). The rp and mc AT (~ 5 g at each depot) samples were collected in dorsal recumbency 15 ± 1 h after LPS infusion. Liver tissue was also sampled for another part of this study at the same time. Both sc AT samples (~ 5 g at each depot) were taken in lateral recumbency 15.5 ± 1 h after LPS infusion. In subsequent evaluations, to avoid scar tissue, sc AT was collected on the other body side (t1) or at least with 5 cm distance from the first sampling point (t2). Each biopsy specimen was immediately flash-frozen in liquid nitrogen (− 196 °C) and stored at − 80 °C until analysis. After surgery, all animals were treated orally with 0.55 mg/kg BW flunixin (Flunidol, CP-Pharma) twice a day for 3 days to reduce post-surgical pain and were assessed for pain on base of a modified pain score [31].

### Morphometric measurements

BW was measured with an electronic scale system designed for large animals (Iconix FX 1, Texas Trading, scale precision: 0.5 kg). At the same time, BCS and CNS were evaluated by two independent observers, each using a scale from 0 to 5 [27, 28]. The average score of the two observers was used for the statistical analyses.

### Blood sample analysis

Plasma glucose concentrations were determined using the glucose-oxidase–peroxidase-method [32]. Serum insulin concentrations were analysed using an immunoradiometric assay (IRMA; Demeditec Diagnostics GmbH, Kiel, Germany; intra-assay CV: 3.12%; inter-assay CV: 5.47%). Serum SAA levels were determined by turbidimetry with test Eiken SAA TIA (Eiken Chemical CO, Tokyo, Japan) which has been validated by [33] and measured with an automated analyser (ABX Pentra 400 analyser; HORIBA Europe GmbH, Oberursel, Germany).

### Determination of mRNA levels in AT

A commercial kit (RNeasy Lipid Tissue Mini Kit and the Qiacube; Qiagen, Hilden, Germany) was used to isolate RNA according to the manufacturer’s protocol. Samples had been stored at − 80 °C pending analysis. The RNA quantity and purity were measured using a spectrophotometer (NanoVue^®^ Plus; Healthcare Biosciences AB, Munich, Germany). RNA quality was determined using an Agilent 2100 Bioanalyzer (Agilent Technologies, Santa Clara, USA).

Two micrograms of RNA were transcribed into cDNA using two master mixes: (1) random primer and dNTP and (2) SuperScript II RT, 5× First Strand Buffer, and 0.1 M DTT (Thermo Fisher Scientific Inc., Schwerte, Germany) and a standard protocol using a thermal cycler (PTC-200, MJ Research, St. Bruno, Canada). cDNA was stored at − 20 °C pending analysis. Genes of interest included markers of inflammation: Cluster of differentiation 68 (CD68), interleukin-1β (IL-1β), interleukin-6 (IL-6) and tumor necrosis factor α (TNFα); marker of lipid metabolism: FABP4 and LPL. As reference, genes 18S ribosomal RNA gene (18S) and ribosomal protein L32 (RPL32) were selected. For genes of interest and RPL32 analysis primer were used (see Additional file 2). For 18S, an RNA probe was used. For quantification of the transcripts, standard curves were generated on every plate with serial dilutions of pooled cDNA of all samples. Additionally, a no-template control was included in each plate. Quantitative polymerase chain reaction was performed using a standard Taqman program (7500 Real Time PCR System, Thermo Fisher Scientific Inc., Schwerte, Germany) with minor modifications (see Additional file 2). Two master mixes were used: Power SYBR Green PCR Master Mix for the genes detected with primers and Taqman Universal PCR Master Mix for *18S* (Thermo Fisher Scientific Inc., Schwerte, Germany). Amplification of specific transcripts was confirmed by melting curve profiles at the end of each PCR. PCR validation parameters which are shown in Additional file 3. The genes of interest were normalised against the geometric mean of the reference genes (18S and RPL32). Their suitability as reference genes was confirmed using BestKeeper [34].

### Statistics

Data analysis was performed using a statistical software program (STATISTICA, StatSoft GmbH, Hamburg, Germany). Data were analysed for normal distribution by the Shapiro–Wilks test. Data for BW, insulin, glucose and SAA were normally distributed. For these parameters ANOVAs with repeated measurements were performed. As post hoc test Fisher’s LSD test was applied. Interaction of time and breed is presented except for the BW × breed interaction. CNS, BCS and mRNA levels are non-parametric data. For these parameters Wilcoxon signed rank test with Bonferroni correction was performed factoring the effects of time. Since in some ponies no AT of rp AT (n = 2), mc AT (n = 3) and neck AT (n = 3) could be sampled at t0 due to the lack of sufficient AT these ponies were excluded from the statistics factoring the effects of time. For breed related differences Mann–Whitney-U test was used. We tested for breed differences at each time point and within one AT depot. To compare different AT depots within a breed Kruskal–Wallis-ANOVA with Bonferroni correction was performed. We only present gene expression differences in AT depots at t2 to focus on AT depot differences in obesity. Statistical analysis of depot differences at all time points is shown in Additional file 4. Statistical significance was accepted at P < 0.05.

## Results

### Morphometric measurements and insulin dysregulation

Ponies and horses significantly increased their BW, BCS and CNS over the BW gaining period (Table 1). In both breed types basal serum insulin concentrations increased over 2-years of BW gain (Table 1). Basal plasma glucose concentrations increased in ponies with BW gain but stayed within reference range [35]. In horses, plasma glucose concentration remained unchanged throughout the study (Table 1). Results of the CGIT were previously published [36]. To summarise the results: at t0 and t1, equines were insulin sensitive based on the results of the CGIT and basal insulin concentrations (Additional file 5). At t2, three out of 19 equines were graded as ID (1 pony and 2 horses). One pony and one horse with ID developed signs of laminitis at different time points in the second year of BW gain. These equines were immediately removed from concentrate feed. At least 1 week after acute signs (pounding digital pulse, lameness) had resolved the sampling procedures were performed in these animals as described above. We collected the samples from the laminitic pony in July, 2017 and of the laminitic horse with its cohort in November, 2017.

**Table 1:** Morphometric measurements (BW, BCS, CNS) and fasted plasma glucose, serum insulin and serum SAA concentrations

Variable	Breed	t0	t1	t2
BW [kg]	Ponies	118 ± 26.7^a^	145 ± 31.9^b^	151 ± 30.3^b^
	Horses	601 ± 44.9^a^	700 ± 43.2^b^	702 ± 44.8^b^
BCS [0–5]	Ponies	2.32 (1.15/3.4)^a^	3.58 (3.4/3.7)^a^	3.94 (3.7/4.15)^b^
	Horses	2.65 (2.05/3.2)^a^	3.6 (3.5/3.6)^b^	3.75 (3.65/3.85)^c^
CNS [0–5]	Ponies	2.5 (0.75/3)^a^	2.75 (2.5/3)^a^	3.5 (3.25/4)^b^
	Horses	2 (1.75/2.25)^a^	2.75 (2.75/3)^b^	3.5 (3.5/4)^c^
Glucose [mmol/L]	Ponies	3.53 ± 0.64^a,#^	3.93 ± 0.38^ab,#^	4.34 ± 0.86^b^
	Horses	4.08 ± 0.21^#^	4.52 ± 0.23^#^	4.41 ± 0.48
Insulin [µU/mL]	Ponies	4.26 ± 1.36^a^	7.93 ± 5.75^ab^	13.9 ± 14.9^b^
	Horses	6.32 ± 2.35^a^	9.3 ± 3.18^ab^	15.1 ± 10.3^b^
SAA [μg/mL]	Ponies	0.35 ± 0.48	0.63 ± 1.24	0.10 ± 0.01
	Horses	0.10 ± 0.01	0.14 ± 0.13	0.10 ± 0.01

t0 = basal measurements, t1 = after 1 year and t2 = after 2 years of hypercaloric diet. Data for BW, glucose, insulin and SAA are expressed as mean ± SD. Data for BCS and CNS are expressed as medians and 25./75. ‰

^a–c^Different superscript letters indicate significant (P < 0.05) differences within rows; ^#^indicates significant (P < 0.05) differences within columns

### Serum amyloid A (SAA)

Basal serum SAA concentrations remained unchanged and within the reference range in the course of the hypercaloric diet in ponies and horses (Table 1). Both laminitic equines had basal serum SAA concentrations within the reference range throughout the study.

### AT mRNA levels

#### Markers of inflammation: influence of BW gain

##### Ponies

**Fig. 2:**
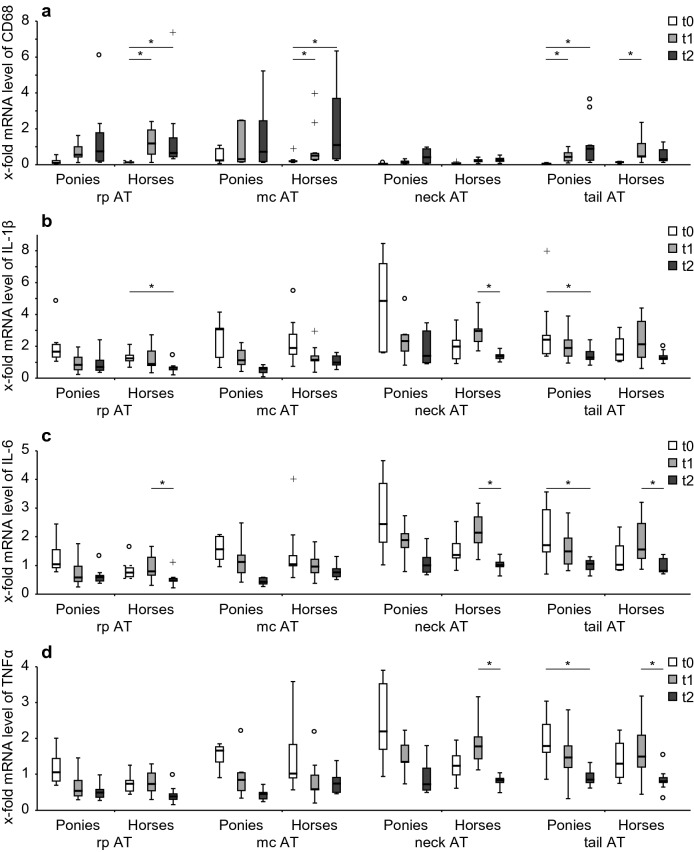
mRNA levels of inflammatory cytokines in AT with BW gain. x-fold mRNA levels of **a***CD68*, **b***IL*-*1β*, **c***IL*-*6* and **d***TNFα* at t0 (white boxes), t1 (grey boxes) and t2 (black boxes) in four different AT depots: rp AT (ponies: n = 8; horses: n = 9), mc AT (ponies: n = 7; horses: n = 9), neck AT (ponies: n = 7; horses: n = 9) and tail AT (ponies: n = 10; horses: n = 9) of ponies and horses. Lines represent the median of the group at each time point, boxes the 25./75. ‰, whiskers the area without outliers, ^o^outliers and ^+^extreme values. *Indicate significant differences (P < 0.05) between different time points within a breed

The *CD68* mRNA levels in tail AT increased from t0 to t1 (P = 0.021) and from t0 to t2 (P = 0.015; Fig. 2a). In tail AT mRNA levels of *IL*-*1β* (P = 0.021; Fig. 2b), *IL*-*6* (P = 0.038; Fig. 2c) and *TNFα* (P = 0.015; Fig. 2c) decreased from t0 to t2. Other AT depots were not affected by BW gain (see also Additional file 6).

##### Horses

*CD68* mRNA levels increased in rp AT from t0 to t1 (P = 0.033) and from t0 to t2 (P = 0.023). An increase in *CD68* mRNA levels was found in mc AT from t0 to t1 (P = 0.023) and from t0 to t2 (P = 0.023). In neck AT *CD68* mRNA levels rose from t0 to t1 (P = 0.045; Fig. 2a). *IL*-*1β* mRNA levels decreased in rp AT from t0 to t2 (P = 0.023) and in neck AT from t1 to t2 (P = 0.023; Fig. 2b). *IL*-*6* mRNA levels decreased from t1 to t2 in rp AT (P = 0.045), neck AT (P = 0.023) and in tail AT (P = 0.023; Fig. 2c). *TNFα* mRNA levels decreased from t1 to t2 in neck AT (P = 0.023) and tail AT (P = 0.045; Fig. 2d, see also Additional file 6).

#### Markers of lipid metabolism: influence of BW gain

##### Ponies

**Fig. 3:**
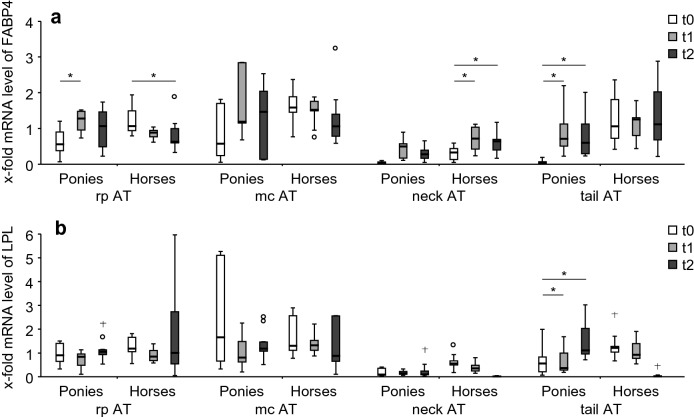
mRNA levels of FABP4 and LPL in AT with BW gain. x-fold mRNA levels of **a***FABP4* and **b***LPL* at t0 (white boxes), t1 (grey boxes) and t2 (black boxes) in four different AT depots: rp AT (ponies: n = 8; horses: n = 9), mc AT (ponies: n = 7; horses: n = 9), neck AT (ponies: n = 7; horses: n = 9) and tail AT (ponies: n = 10; horses: n = 9) of ponies and horses. Lines represent the median of the group at each time point, boxes the 25./75. ‰, whiskers the area without outliers, ^o^outliers and ^+^extreme values. *Indicate significant differences (P < 0.05) between different time points within a breed

*FABP4* mRNA level increased in rp AT from t0 to t1 (P = 0.035). In tail AT *FABP4* and *LPL* mRNA levels increased from t0 to t1 (P = 0.015; P = 0.015) and from t0 to t2 (P = 0.015; P = 0.021; Fig. 3a, b).

##### Horses

*FABP4* mRNA levels decreased in rp AT from t0 to t2 (P = 0.045) but increased in neck AT from t0 to t1 (P = 0.023) and from t0 to t2 (P = 0.023). No effect of BW gain was observed on *LPL* mRNA level in the different AT tissues (Fig. 3a, b).

#### Markers of inflammation: influence of breed type

Horses had higher *CD68* mRNA levels at t0 in neck AT (P = 0.044) and tail AT (P = 0.002) than ponies. *IL*-*1β* mRNA levels of horses were higher at t2 in the mc AT (P = 0.006) but lower at t0 in neck AT (P = 0.026) than ponies. In mc AT *IL*-*6* mRNA levels were higher in horses than in ponies at t2 (P = 0.005). At t0 *IL*-*6* and *TNFα* mRNA levels of horses were lower in rp AT (P = 0.030 and P = 0.024) and in neck AT (P = 0.034 and P = 0.020) compared to ponies.

#### Markers of lipid metabolism: influence of breed type

In general, horses had higher *FABP4* and *LPL* mRNA levels compared to ponies in neck AT and tail AT at all time points. At t0 rp AT of horses had higher *FABP4* mRNA levels compared to ponies (P = 0.011). In mc AT higher *LPL* mRNA levels were found in horses compared to ponies at t2 (P = 0.045).

#### Markers of inflammation: AT depot dependent differences at t2

**Fig. 4:**
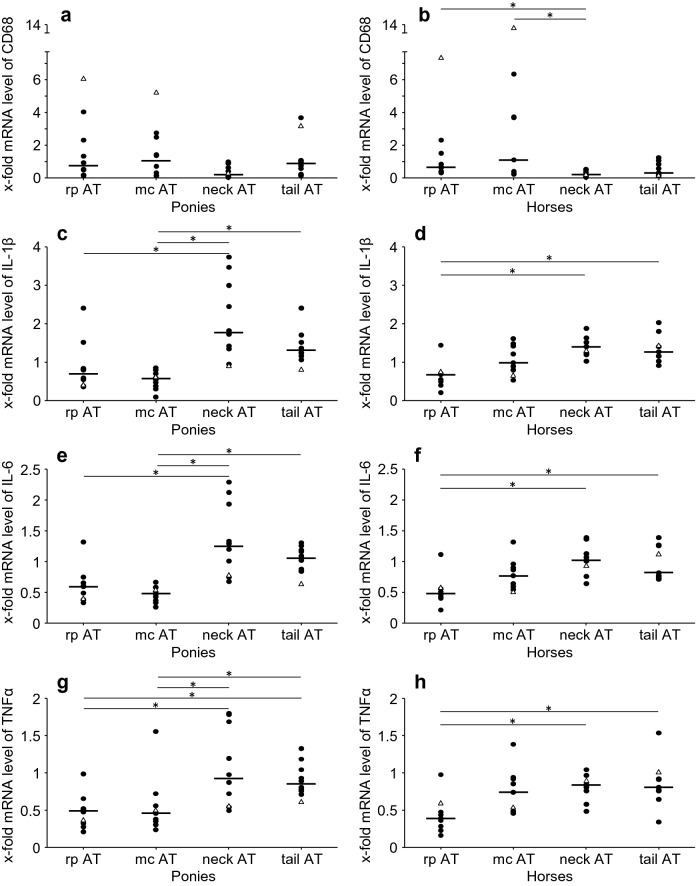
mRNA levels of inflammatory are legendsytokines: comparison of AT depots. x-fold mRNA levels of (**a**, **b**) *CD68*, (**c**, **d**) *IL*-*1β*, (**e**, **f**) *IL*-*6* and (**g**, **h**) *TNFα* at t2 in four different AT depots of ponies (n = 10) and horses (n = 9). Dots represent single values of equines and lines represent the median of the breed. Values of laminitic equines are shown as triangles. *Indicate significant differences (P < 0.05) between different AT depots within a breed

Depot specific differences at t2 are shown in Fig. 4. Rp AT and mc AT had higher *CD68* mRNA levels (Fig. 4a, b) but neck AT and tail AT showed higher mRNA levels of *IL*-*1β*, *IL*-*6* and *TNFα* (Fig. 4c–h).

#### Markers of lipid metabolism: AT depot dependent differences at t2 (see also Additional file 6)

**Fig. 5:**
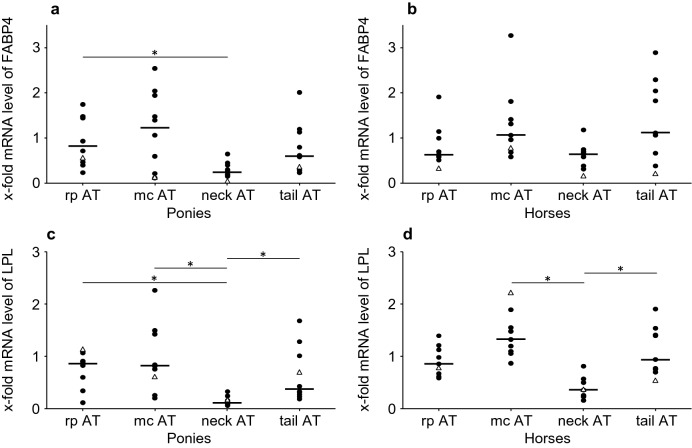
mRNA levels of FABP4 and LPL: comparison of AT depots. x-fold mRNA levels of **a**, **b***FABP4* and **c**, **d***LPL* at t2 in four different AT depots of ponies (n = 10) and horses (n = 9). Dots represent single values of equines, and lines represent the median of the group. Values of laminitic equines are shown as triangles. *indicate significant differences (P < 0.05) between different AT depots within a breed

AT depot specific differences in FABP4 and LPL mRNA levels are shown in Fig. 5.

## Discussion

This study provides further insight into the changes in AT inflammation expression patterns associated with long-term BW gain in ponies and horses. To the authors’ knowledge this is the first study investigating the effect of long-term BW gain on inflammatory cytokines in multiple AT depots of two breed types over time under standardized housing and feeding conditions. We had hypothesized that there would be an increase in inflammatory cytokines, particularly in sc AT, over the period of BW gain in combination with a low endotoxin stimulus. The equines showed an increased BW, BCS and CNS, but 16 out of 19 equines showed no ID within 2 years of BW gain. The results according to ID were previously published by our research group [36]. An interesting finding was that the main BW gain was observed in the first year of hypercaloric diet in both breeds. Lower BW gain was found in the second year despite the same level of hypercaloric intake. In contrast BCS and CNS increased significantly in the second year of feeding period in both breeds. One explanation might be related to an increase in fat mass in expense to a loss in muscle mass due to lower physical activity during periods of turnout. From the present data, the reasons for these findings remained unexplained since muscle metabolism was not in the focus of the study. The lack in knowledge about muscle metabolism in the state of increasing obesity should be addressed in future studies.

A main limitation of this study was the determination of mRNA levels of selected inflammatory parameters without analysis of the corresponding protein concentrations. However, the repeated measurements indicated the direction of inflammatory cytokine levels during BW gain. Also, a control group receiving an isocaloric diet would have been a powerful verification of our results. In most previous equine studies, the comparison of lean (control) and obese animals were restricted to animals kept under different feeding and management conditions. To compensate for the missing control group, we used a repeated measurement study design in order to reduce variation. In consequence, each animal was used as its own control. To exclude any seasonal effect, we took the samples at the same time of the year.

We used a low dosage of LPS 15 h before sampling to trigger a low level of tissue inflammation [37–39]. In that context, Tadros et al. [37] described comparable mRNA levels of proinflammatory cytokines in the liver tissue in horses affected by EMS compared to healthy horses 240 up to 360 min after LPS infusion. However, in EMS horses, whole blood cytokine expression was prolonged after an infusion of 20 ng/kg BW LPS compared to healthy horses. These data support the hypothesis that EMS horses did not develop higher levels of inflammatory cytokines per se but the inflammatory response to a trigger seemed to be different. To aim these differences with increasing BW, we used a moderate endoxin trigger to induce a more distinguishable proinflammatory response.

Since systemic inflammation in obese equines is still under debate [10, 11, 14], we determined serum SAA concentrations before endotoxin stimulus, which is one of the preferred acute phase proteins in equines to detect inflammation [40]. Plasma SAA has been reported to have a positive correlation with BCS and insulin concentrations in a mixed population of light-breed horses with unclear duration of being obese [9]. However, the plasma SAA concentrations of the above-mentioned study population were far below the threshold for indicating inflammation. Our findings showed unaltered serum SAA concentrations with increasing obesity. It remains still controversial if obesity in equines is related to inflammation. The outcome of our study might be limited to the fact that our equines were only moderately obese at the end of the study and had undergone only 2 years of BW gain.

Macrophage invasion in AT has been described to occur with increasing obesity in both mice and humans [5, 41]. Therefore, it is not surprising that *CD68* mRNA levels, a well-known macrophage marker, increased with BW gain in the present study. We used CD68 as a marker for macrophage invasion, which indicates the number of macrophages in equines and humans [42, 43]. Obese humans show higher macrophage abundance in abdominal AT depots compared to sc AT depots [44]. These findings are in accordance with the results of the present study, as horses had higher *CD68* mRNA levels in rp and mc AT compared to neck AT. Our results also support the morphologic observations of Siegers et al. [45], who found a greater expandability of rp AT in overfed pony mares. The authors linked the fat accumulation in rp AT, based on ultrasound measurements, to a greater impact on metabolic health in Shetland ponies. Interestingly, *CD68* mRNA levels were 8.2- and 11.3-fold higher in rp AT and 5.0- or 12.5-fold higher in mc AT of the laminitic pony and horse, respectively, compared to the median for their breed types at t2 after endotoxin stimulus. A similar relationship between macrophage invasion of AT and obesity comorbidities has been reported in humans [46] and a correlation seems to be likely in equines based on our findings. Although the outcome is limited by the small number of animals affected in this study, further research is encouraged. No breed type related differences were found in obese ponies and horses. Controversy exists about the low state of inflammation based on cytokines reported in obese horses and ponies [10, 11, 14]. In a spot sample study in a group of light breed mares BCS correlated positively with inflammatory cytokines [8]. Contrariwise, no correlation of BCS and inflammatory cytokines was found in a mixed population of light breed horses [9]. In BW gaining studies, feeding horses a hypercaloric diet for 24 and 20 weeks, respectively, had no effect on serum TNFα levels [11, 47]. This is in accordance with our results. In our study *IL*-*1β*, *IL*-*6* and *TNFα* mRNA levels remained unchanged in most AT depots even though an endotoxin stimulus was used. These findings may indicate that, in contrast to humans, equine obesity is not strictly associated with increasing inflammatory cytokine levels in AT. Interestingly, Waller et al. [14] found higher TNFα levels in abd AT of insulin resistant mares compared to insulin sensitive mares that were kept under similar feeding and management practices. Additionally, a group of obese Welsh ponies with EMS (BCS: 8.37 ± 2.44 on a scale of 1 to 9) had higher serum TNFα and AT IL-6 concentrations compared to obese Welsh ponies without EMS (BCS: 7.25 ± 0.46 on a score of 9) [12]. From these data, it can be concluded that obesity does not necessarily lead to a low grade of inflammation. In future studies, a better understanding between metabolically ‘healthy’ from ‘unhealthy’ obese individuals needs further clarification.

Different AT depots produce a varying number of inflammatory cytokines in humans and horses [15, 16]. Contrary to the *CD68* mRNA levels, inflammatory cytokines *IL*-*1β*, *IL*-*6* and *TNFα* mRNA were higher in sc AT compared to the abd AT after BW gain in the present study. In agreement with our results, Burns et al. [48] reported higher *IL*-*6* and *IL*-*1β* mRNA levels in neck AT compared to abd AT depots in light breed mares. This might be an explanation for the link between the abundance of nuchal AT and the likelihood of being IR [28]. Breed related differences were only detected in mesocolon AT with higher expression levels of inflammatory markers in obese horses compared to obese ponies. This contrasts our hypothesis as we expected higher inflammatory markers in Shetland ponies than in Warmblood horses. As these findings were only present in one AT depot, the metabolic consequences remain unclear and need further elucidation.

Obesity and its comorbidities do not only influence inflammation, but also lipid metabolism in horses [9, 49]. Therefore, we measured the expression of genes affecting lipid metabolism in AT. *FABP4* is expressed in AT and its function is related to the transport of long-chain fatty acid inside adipocytes. Circulating FABP4 levels are increased in obesity and metabolic diseases in humans and rats [50–52]. In contrast, *FABP4* mRNA levels were decreasing with the degree of obesity in sc AT and visceral AT of humans [26]. In the present study, both breeds had an increase in *FABP4* mRNA levels with BW gain in neck and tail AT, respectively. The reason might be an increased turnover of fatty acids in sc AT with BW gain in equines. *FABP4* mRNA levels were higher in rp AT of ponies compared to neck AT at t2. This agrees with findings of Siegers et al. [45], who suggested a higher uptake of fatty acids into rp AT than sc AT with BW gain.

The enzyme LPL catalyzes the hydrolysis, absorption and therefore the clearance of triacylglycerides from the blood [21]. In women, higher LPL mRNA levels were found in sc AT depots compared to omental AT [53]. In contrast to observations in humans, our equines had higher *LPL* mRNA levels in abd AT compared to sc AT depots at t2, which may indicate a higher capacity of fatty acids in abd AT. This hypothesis is supported by higher *FABP4* levels in rp AT compared to neck AT. *LPL* mRNA levels in sc AT were lower in obese humans with diabetes compared to healthy individuals but not to obese patients without diabetes [22]. This is in accordance with observations that unhealthy (with comorbidities) obese humans primary distribute fat into visceral AT and develop ectopic fat accumulation in liver and muscle [1]. In the present study, ponies had higher *LPL* mRNA levels in tail AT after the long-term intake of a hypercaloric diet. After BW gain *LPL* mRNA levels were higher in AT of horses compared to ponies. In another part of this study we found higher *LPL* mRNA abundance in liver tissue in the same ponies compared to horses at t2 [54]. It can be speculated that the horses still had the capacity to store fatty acids in AT whereas ponies already started to store fatty acids in ectopic tissues like the liver.

## Conclusions

Our study provides evidence for higher macrophage infiltration into AT with the development of obesity in equines when they are fed 200% of their recommended energy intake for 2 years and in response to a low dose endotoxin stimulus. The macrophage invasion was more pronounced in abd AT compared to sc AT in both breeds. However, the grade of macrophage invasion might predispose susceptible equines to obesity related comorbidities. The question remains open whether macrophages in AT of obese equines need a specific trigger for activation to enhance inflammatory cytokine production. Contrariwise, subcutaneous adipose tissue expressed more pro-inflammatory cytokines compared to intra-abdominal adipose tissue.

In horses, higher *FABP4* and *LPL* mRNA levels in selected AT depots may indicate a higher fat storage capacity in AT than in ponies. In contrast, obese ponies may store fatty acids to an earlier onset of obesity in ectopic tissues like the liver. The differences in lipid storage might be associated with a higher susceptibility to obesity-related comorbidities in ponies compared to horses.

## Supplementary information

**Additional file 1.** Daily dietary intake and ration composition during BW gaining period for ponies and horses.

**Additional file 2.** Primer sequences and PCR protocoll used to analyze the levels of the genes of interest and reference genes.

**Additional file 3.** RT-qPCR validation parameters.

**Additional file 4.** Comparison of mRNA expression levels between AT depots at t0, t1 and t2.

**Additional file 5.** Original data of BW, BCS, CNS, SAA, Glucose and Insulin.

**Additional file 6.** Original data of AT gene expression.

## Data Availability

All data generated or analysed during this study are included in this published article and its additional information files.

## Abbreviations

Abd: Abdominal
AT: Adipose tissue
BCS: Body condition score
BW: Body weight
CD68: Cluster of differentiation 68
CNS: Crest neck score
EMS: Equine metabolic syndrome
FABP4: Fatty acid binding protein
ID: Insulin dysregulation
IL: Interleukin
IR: Insulin resistance
LPL: Lipoprotein lipase
mc: Mesocolonial
neck AT: Adipose tissue of the neck crest
rp: Retroperitoneal
sc: Subcutaneous
tail AT: Adipose tissue lateral to the tail head
TNF: Tumor necrosis factor
