# Supplementary material for: The influence of equine body weight gain on inflammatory cytokine expressions of adipose tissue in response to endotoxin challenge
Source: Acta Vet Scand. 2020 Apr 22;62:17. doi: 10.1186/s13028-020-00515-5 (PMC7178607; doi:10.1186/s13028-020-00515-5)
Supplement: Supplementary file 1 — Additional file 1. Daily dietary intake and ration composition during BW gaining period for ponies and horses. [file 13028_2020_515_MOESM1_ESM.docx]

**Additional Table 1. Daily dietary intake and ration composition during BW gaining period for ponies and horses.**

| Variable | Component | Ponies | | Horses | |
| --- | --- | --- | --- | --- | --- |
| Feed intake  [kg DM / 100 kg BW] | Meadow hay | 1.95 | ± 0.16 | 1.53 | ± 0.13 |
|  | Compound feed | 0.54 | ± 0.08 | 0.48 | ± 0.07 |
| Nutrient intake  [% of dry matter intake] | Crude protein | 9.07 | ± 1.85 | 9.20 | ± 1.80 |
|  | Crude fibre | 29.1 | ± 2.62 | 28.5 | ± 2.50 |
|  | Crude fat | 4.42 | ± 0.42 | 4.70 | ± 0.41 |
|  | Sugar | 9.91 | ± 1.07 | 9.45 | ± 0.77 |
|  | Starch | 7.45 | ± 0.11 | 8.20 | ± 0.12 |

Data are expressed as the mean ± SD
