# Supplementary material for: The influence of equine body weight gain on inflammatory cytokine expressions of adipose tissue in response to endotoxin challenge
Source: Acta Vet Scand. 2020 Apr 22;62:17. doi: 10.1186/s13028-020-00515-5 (PMC7178607; doi:10.1186/s13028-020-00515-5)
Supplement: Supplementary file 2 — Additional file 2. Primer sequences and PCR protocoll used to analyze the levels of the genes of interest and reference genes. [file 13028_2020_515_MOESM2_ESM.docx]

**Additional table 2. Primer sequences and PCR protocoll used to analyze the levels of the genes of interest and reference genes.**

|  | Forward (5´-3´) | Reverse (5´-3´) |
| --- | --- | --- |
| RPL32[1] | AGCCATCTACTCGGCGTCA | TCCAATGCCTCTGGGTTTC |
| CD68[2] | CTTTGGGCCAAGTTTCTCTTGT | AAGAGGCCGAGGAGGATCAG |
| IL-1β ^#^ | CGGCAATGAGAATGACCTGT | GCTTCTCCACAGCCACAATG |
| IL-6[2] | CCACCTCAAATGGACCACTACTC | TTTTCAGGGCAGAGATTTTGC |
| TNFα[3] | AAAGGACATCATGAGCACTGAAAG | GGGCCCCCTGCCTTCT |
| FABP-4^#^ | GCATTTGTAGGCACCTGGAA | TGGTGATTACGTCCCCATTCA |
| LPL ^#^ | ATTGTGGTGGACTGGCTGT | GCTCCAAGGCTGTATCCCAA |

^#^ Designed using http://primer3.ut.ee/. The specific equine cDNA sequences were provided by <http://www.ensembl.org/index.html> and the generated primers were validated in <http://eu.idtdna.com/calc/analyzer> to confirm the absence of hairpins, homodimers and heterodimers. The designed primers were created with two different modifications for each gene of interest and the more suitable primer was selected in preliminary tests. Primers were synthesized by biomers.net GmbH.

**Temperature profiles PCR**

UNG-Verdau: 50°C for two minutes

AmpliTaq Gold activation: 95°C for 10 minutes

45 Cycles: denaturation: 95°C for 15 seconds

annealing/elongation: 60°C for one minute

References

1. Bogaert L, van Poucke M, Baere C de, Peelman L, Gasthuys F, Martens A. Selection of a set of reliable reference genes for quantitative real-time PCR in normal equine skin and in equine sarcoids. BMC Biotechnol. 2006; 6: 24. doi: 10.1186/1472-6750-6-24.

2. Ungru J, Blüher M, Coenen M, Raila J, Boston RC, Vervuert I. Effects of body weight reduction on blood adipokines and subcutaneous adipose tissue adipokine mRNA expression profiles in obese ponies. Vet Rec. 2012; 171: 528. doi: 10.1136/vr.100911.

3. Figueiredo MD, Salter CE, Andrietti ALP, Vandenplas ML, Hurley DJ, Moore JN. Validation of a reliable set of primer pairs for measuring gene expression by real-time quantitative RT-PCR in equine leukocytes. Vet. Immunol. Immunopathol. 2009; 131: 65–72. doi: 10.1016/j.vetimm.2009.03.013.
