# Supplementary material for: The influence of equine body weight gain on inflammatory cytokine expressions of adipose tissue in response to endotoxin challenge
Source: Acta Vet Scand. 2020 Apr 22;62:17. doi: 10.1186/s13028-020-00515-5 (PMC7178607; doi:10.1186/s13028-020-00515-5)
Supplement: Supplementary file 4 — Additional file 4. Comparison of mRNA expression levels between AT depots at t0, t1 and t2. [file 13028_2020_515_MOESM4_ESM.docx]

**Table S4: Comparison of mRNA expression levels between AT depots at t0, t1 and t2.**

| Variable | Time point | Retroperitoneal AT | | Mesocolonial AT | | Sc AT of nuchal crest | | Sc AT of the tail head | | P^*^ |
| --- | --- | --- | --- | --- | --- | --- | --- | --- | --- | --- |
|  |  |  | |  | |  | |  | |  |
| Ponies |  |  | |  | |  | |  | |  |
| CD68 | t0 | 0.10 | (0.08/0.25)^ab^ | 0.25 | (0.19/0.92)^a^ | 0.05 | (0.04/0.06)^b^ | 0.05 | (0.03/0.07)^b^ | 0.001 |
|  | t1 | 0.51 | (0.30/0.84) | 0.21 | (0.05/0.93) | 0.12 | (0.06/0.23) | 0.44 | (0.18/0.69) | 0.048 |
|  | t2 | 0.75 | (0.18/2.30) | 1.04 | (0.15/2.48) | 0.21 | (0.08/0.63) | 0.89 | (0.21/1.09) | 0.326 |
| IL-1β | t0 | 1.68 | (1.30/2.15) | 3.07 | (1.28/3.16) | 4.86 | (1.61/7.23) | 2.42 | (1.51/2.72) | 0.112 |
|  | t1 | 0.92 | (0.60/1.76) | 1.01 | (0.78/1.79) | 2.39 | (1.67/3.39) | 1.90 | (1.36/2.44) | 0.045 |
|  | t2 | 0.70 | (0.41/0.83)^ac^ | 0.58 | (0.39/0.70)^a^ | 1.77 | (1.35/2.99)^b^ | 1.31 | (1.16/1.71)^bc^ | 0.000 |
| IL-6 | t0 | 1.04 | (0.90/1.57)^a^ | 1.56 | (1.20/2.02)^ab^ | 2.45 | (1.79/3.88)^b^ | 1.71 | (1.46/2.97)^ab^ | 0.034 |
|  | t1 | 0.65 | (0.48/1.28)^a^ | 0.99 | (0.68/1.37)^ab^ | 1.86 | (1.61/2.13)^b^ | 1.49 | (1.04/1.97)^ab^ | 0.019 |
|  | t2 | 0.59 | (0.40/0.65)^ac^ | 0.48 | (0.37/0.58)^a^ | 1.25 | (0.79/1.93)^b^ | 1.05 | (0.84/1.19)^bc^ | 0.000 |
| TNFα | t0 | 1.06 | (0.80/1.46)^a^ | 1.67 | (1.33/1.78)^ab^ | 2.20 | (1.68/3.55)^b^ | 1.79 | (1.60/2.41)^ab^ | 0.020 |
|  | t1 | 0.59 | (0.40/1.05) | 0.81 | 0.54/1.05) | 1.36 | (1.33/1.83) | 1.47 | (1.18/1.81) | 0.045 |
|  | t2 | 0.49 | (0.32/0.52)^a^ | 0.46 | (0.35/0.56)^a^ | 0.93 | (0.56/1.69)^b^ | 0.85 | (0.75/1.05)^b^ | 0.001 |
| FABP4 | t0 | 0.56 | (0.37/0.91)^a^ | 0.58 | (0.23/1.71)^a^ | 0.03 | (0.01/0.07)^b^ | 0.05 | (0.01/0.08)^b^ | 0.000 |
|  | t1 | 1.28 | (0.89/1.49)^a^ | 1.24 | (1.15/2.38)^a^ | 0.18 | (0.14/0.54)^b^ | 0.72 | (0.50/1.13)^ab^ | 0.001 |
|  | t2 | 0.82 | (0.50/1.46)^a^ | 1.23 | (0.31/1.83)^ab^ | 0.24 | (0.18/0.37)^b^ | 0.60 | (0.29/1.14)^ab^ | 0.021 |
| LPL | t0 | 1.01 | (0.53/2.75)^a^ | 0.89 | (0.64/2.57)^a^ | 0.02 | (0.01/0.05)^b^ | 0.03 | (0.02/0.5)^b^ | 0.000 |
|  | t1 | 0.78 | (0.55/1.28)^a^ | 0.99 | (0.68/1.69)^a^ | 0.09 | (0.45/0.27)^b^ | 0.57 | (0.20/0.84)^ab^ | 0.001 |
|  | t2 | 0.86 | (0.65/0.90)^a^ | 0.82 | (0.65/1.28)^a^ | 0.11 | (0.10/0.16)^b^ | 0.64 | (0.27/1.01)^a^ | 0.000 |
|  |  |  |  |  |  |  |  |  |  |  |
| Horses |  |  |  |  |  |  |  |  |  |  |
| CD68 | t0 | 0.13 | (0.10/0.16)^ab^ | 0.19 | (0.16/0.23)^a^ | 0.08 | (0.07/0.09)^b^ | 0.14 | (0.11/0.16)ab | 0.002 |
|  | t1 | 1.18 | (0.56/1.96)^a^ | 0.51 | (0.27/0.66)^ab^ | 0.19 | (0.15/0.30)^b^ | 0.46 | (0.40/1.21)^ab^ | 0.008 |
|  | t2 | 0.65 | (0.41/1.52)^a^ | 1.09 | (0.29/3.72)^a^ | 0.21 | (0.17/0.38)^b^ | 0.32 | (0.20/0.86)^ab^ | 0.007 |
| IL-1β | t0 | 1.26 | (1.07/1.48) | 1.90 | (1.48/2.79) | 2.00 | (1.19/2.42) | 1.49 | (1.09/2.50) | 0.188 |
|  | t1 | 0.89 | (0.76/1.74)^a^ | 1.17 | (1.06/1.43)^a^ | 2.96 | (2.27/3.12)^b^ | 2.13 | (1.29/3.60)^ab^ | 0.002 |
|  | t2 | 0.67 | (0.49/0.71)^a^ | 0.99 | (0.80/1.43)^ab^ | 1.40 | (1.21/1.51)^b^ | 1.27 | (1.16/1.44)^b^ | 0.003 |
| IL-6 | t0 | 0.76 | (0.59/0.94)^a^ | 1.04 | (0.97/1.36)^ab^ | 1.37 | (1.25/1.78)^b^ | 1.03 | (0.84/1.70)^ab^ | 0.016 |
|  | t1 | 0.80 | (0.65/1.30)^a^ | 0.97 | (0.73/1.24)^a^ | 2.15 | (1.78/2.71)^b^ | 1.56 | (1.23/2.49)^ab^ | 0.001 |
|  | t2 | 0.48 | (0.43/0.56)^a^ | 0.77 | (0.59/0.90)^ab^ | 1.02 | (0.94/1.13)^b^ | 0.82 | (0.76/1.26)^b^ | 0.001 |
| TNFα | t0 | 0.74 | (0.52/0.84)^a^ | 1.02 | 0.89/1.85)^ab^ | 1.24 | (0.97/1.53)^ab^ | 1.30 | (0.90/1.88)^b^ | 0.017 |
|  | t1 | 0.74 | (0.53/1.04)^a^ | 0.59 | (0.56/0.99)^a^ | 1.78 | (1.42/2.06)^b^ | 1.50 | (1.19/2.10)^ab^ | 0.001 |
|  | t2 | 0.39 | (0.29/0.47)^a^ | 0.74 | (0.48/0.92)^ab^ | 0.84 | (0.76/0.90)^b^ | 0.81 | (0.76/0.92)^b^ | 0.017 |
| FABP4 | t0 | 1.07 | (0.93/1.50)^a^ | 1.59 | (1.44/1.90)^a^ | 0.33 | (0.12/0.45)^b^ | 1.06 | (0.71/1.82)^a^ | 0.000 |
|  | t1 | 0.88 | (0.76/0.95)^a^ | 1.53 | (1.48/1.77)^b^ | 0.72 | (0.41/1.05)^a^ | 1.25 | (0.79/1.31)^ab^ | 0.002 |
|  | t2 | 0.63 | (0.89/1.00) | 1.07 | (0.77/1.41) | 0.65 | (0.39/0.70) | 1.12 | (0.67/2.04) | 0.059 |
| LPL | t0 | 1.06 | (0.93/1.15)^ab^ | 1.19 | (1.06/1.48)^a^ | 0.11 | (0.10/0.25)^b^ | 1.12 | (0.94/2.05)^a^ | 0.002 |
|  | t1 | 1.19 | (1.04/1.68)^ab^ | 1.31 | (1.10/2.58)^a^ | 0.57 | (0.49/0.69)^b^ | 1.22 | (1.03/1.29)^ab^ | 0.004 |
|  | t2 | 0.85 | (0.67/1.12)^ab^ | 1.33 | (1.10/1.55)^a^ | 0.36 | (0.24/0.50)^b^ | 0.93 | (0.76/1.41)^a^ | 0.000 |
|  |  |  |  |  |  |  |  |  |  |  |

t0 = basal measurements, t1 = after one year and t2 = after two ears of hypercaloric diet. Data are expressed as median (25./75. ‰);^*^P-value of Kruskal-Wallis-ANOVA; ^a-b^Different superscript letters indicate significant (P < 0.05) differences within rows with post hoc Bonferroni correction.
